# Air Pollution: Possible Interaction between the Immune and Nervous System?

**DOI:** 10.3390/ijerph192316037

**Published:** 2022-11-30

**Authors:** Melania Maria Serafini, Ambra Maddalon, Martina Iulini, Valentina Galbiati

**Affiliations:** Department of Pharmacological and Biomolecular Sciences (DiSFeB), Università degli Studi di Milano, 20133 Milan, Italy

**Keywords:** air pollution, immune system, skin, central nervous system, microbiota, hygiene hypothesis

## Abstract

Exposure to environmental pollutants is a serious and common public health concern associated with growing morbidity and mortality worldwide, as well as economic burden. In recent years, the toxic effects associated with air pollution have been intensively studied, with a particular focus on the lung and cardiovascular system, mainly associated with particulate matter exposure. However, epidemiological and mechanistic studies suggest that air pollution can also influence skin integrity and may have a significant adverse impact on the immune and nervous system. Air pollution exposure already starts in utero before birth, potentially causing delayed chronic diseases arising later in life. There are, indeed, time windows during the life of individuals who are more susceptible to air pollution exposure, which may result in more severe outcomes. In this review paper, we provide an overview of findings that have established the effects of air pollutants on the immune and nervous system, and speculate on the possible interaction between them, based on mechanistic data.

## 1. Air Pollution: State of the Art

Air pollution can be defined broadly as the introduction of chemicals, particulate matter or biological materials into the atmosphere, that may cause harm or discomfort to humans and other living organisms, or cause damage to the environment [1,2,3]. Air pollutants may be categorized as primary air pollutants, which are directly emitted to the atmosphere, or secondary air pollutants, formed in the atmosphere (e.g., from the oxidation and transformation of primary emissions). Air pollution can derive from two different sources: indoor and outdoor. Indoor air pollution is produced first by penetration of outdoor pollutants, second by activities such as cooking and cooling/heating, and finally by building products, which differ accordingly to the construction materials used (for an extensive review on indoor air quality, see [4,5]). In this review, we will focus on outdoor air pollution. To deepen the knowledge about air pollution effects on the human beings is mandatory, since the World Health Organization (WHO) reported that the majority of the population worldwide is exposed to air containing higher levels of pollutants compared to the guidelines [6]. What is clearly known is that air pollution exposure represents an environmental risk factor for human health and its impact is relevant due to the large number of individuals affected, especially in densely populated areas [7]. In fact, a relationship between long-term urbanization and outdoor air pollution has been demonstrated [8]. While in the developed countries there is a common trend of decline in pollutant levels, in the low and middle-income nations, the situation is various: there are countries (e.g., India and China) where air pollution levels have risen, with exposure overcoming safety standards, and nations (e.g., Mexico) where levels have declined [9].

These issues posed by outdoor air pollutants have increased the amount of research conducted to dissect the mechanisms by which they exert their toxicity. While the majority of the studies have focused on the effects on the general health status, only some of them have investigated the effects on the immune and nervous systems, which instead are of great relevance.

Atmospheric pollutants typically include particulate matter (PM), ozone (O_3_), nitrogen oxide (NO_x_), polycyclic aromatic hydrocarbons (PAHs) (in particular benzo-a-pyrene-BaP), sulphur oxide (SO_x_), carbon monoxide (CO), benzene, and several metals such as manganese (Mn), arsenic (As), cadmium (Cd), lead (Pb), mercury (Hg), nickel (Ni), aluminium (Al), and vanadium (V) generated by vehicular traffic, industrial emissions, and biomass burning [10,11]. 

The PM component of air pollution is characterized by its size and aerodynamic properties: coarse particle with aerodynamic diameter of 2.5 to 10 µm (PM_10_), fine particles of less than 2.5 µm (PM_2.5_), and ultrafine particulate matter (UFPM) of less than 0.1 µm, also called nanosized particulate matter or PM_0.1_ [12,13]. The incomplete combustion of fuel and diesel engine oil leads to the formation of gaseous air pollutants (CO, NO, NO_2_, aldehydes), as well as solid particles including diesel exhaust particles (DEPs). DEPs are composed of a carbon core upon which high-molecular weight organic chemical components (CO, NO_x_, SO_2_, hydrocarbons) and heavy metals deposit. Diesel exhaust (DE) is a complex mixture and, in this review, we will generally refer to DE when the solid or gaseous nature of the pollutant is not specified in the original research paper.

Recently, there has been a dramatic increase in the prevalence of non-communicable disease, such as cardiovascular disease, metabolic disease, cancer, chronic obstructive pulmonary disease, inflammatory bowel diseases, and allergic diseases [14]. Migration from the countryside to cities, and from economically backward to economically developed countries has exposed migrants and their families to a new set of potentially harmful pollutants and allergens and changed their housing conditions, diet and access to medical services [10,15]. Recent studies and research programs consistently show that harmful health effects induced by air pollution can be observed also to very low concentration levels, with no observable thresholds below which exposure can be considered safe [16,17,18]. WHO produces evidence regarding the linkage of air pollution to specific diseases and the overall objective of the global air quality guidelines published in 2021 is to offer quantitative health-based recommendations through the assessment of the air quality guideline (AQG) levels. AQC levels are not legally binding standards but nevertheless represent an evidence-informed tool, which can be used to inform legislation and policy [19]. The document reported specific recommendations on AQG levels for the pollutants PM_2.5_, PM_10_, ozone, nitrogen dioxide, sulfur dioxide, and carbon monoxide (Table 1). A region-specific analysis related to the global deaths attributable to ambient PM_2.5_ air pollution and the correspondent reduction through achievement of the recommended AQG level demonstrate that each reduction in the outdoor concentrations of key air pollutants brings health benefits to the surrounding population, even in places, which already have low pollution concentrations [19]. 

The route of exposure to air pollutants is mainly through inhalation, ingestion, and skin contact; therefore, these systems may be affected. In the past decade, it was taken for granted that PM exposure constitutes a risk factor for cardiovascular diseases [20]. However, epidemiological studies have raised the concern about the potential impact of air pollution on the central nervous system (CNS) outcomes, including decreased cognitive functions in children, adults, and the elderly, neurodevelopment diseases, depressive symptoms, olfactory dysfunctions, auditory deficits, and other adverse neuropsychological effects [21,22]. The WHO International Agency for Research on Cancer concluded, already in 2013, that outdoor air pollution might be carcinogenic to humans, going on the assumption that hazard-based mixtures containing carcinogens are necessarily carcinogenic themselves, with the PM component of air pollution most closely associated with increased cancer incidence, especially lung cancer [23]. In this context, the time window of exposure is of great relevance. In fact, during the life of individuals, some periods (e.g., childhood and old age) are more susceptible to air pollution, possibly resulting in more severe outcomes.

The European Environment Agency suggests that exposure to air pollutants in early life can significantly affect childhood development and could trigger diseases later in life [24]. It has also been reported that during pregnancy, in utero exposure to air pollutants enhances adverse birth outcomes, such as reduced fetal growth, pre-term birth, and spontaneous abortions [25]. All environmental exposures, from in utero to adult life, may lead to epigenetic changes and the level of DNA methylation varies significantly with different types of air pollution [26,27]. As well, as reported by the WHO, exposure to air pollution may lead to adverse health effects, such as premature mortality and morbidity. Mortality reflects a reduction in life expectancy owing to premature death as a result of exposure to air pollution. Differently, morbidity relates to the occurrence of illness and years lived with a disease or disability, ranging from subclinical effects and symptoms (e.g., inflammation) to chronic conditions that may require hospitalization. Even less severe effects might have considerable public health implications because air pollution affects the whole population daily [28].

The mechanisms underneath the health effects of air pollution, due to its complex composition, are mainly referable to a direct or indirect reactive oxygen and nitrogen species (ROS, RNS) production causing oxidative stress with the activation of inflammatory pathways (e.g., Nrf2, MAPK, NF-κB) that lead to an inflammatory status characterized by cytokine production and immune cell activation [29], but also by epigenetic mechanisms, such as change in histone tail modifications, microRNA (miRNA) expression, and DNA methylation, that contribute to the development and maintenance of inflammation [30].

**Figure 1:**
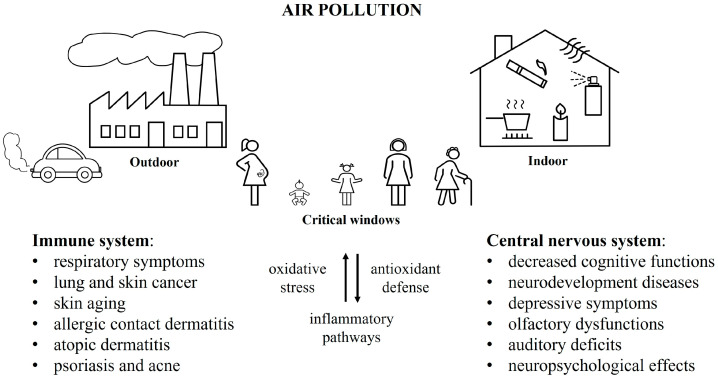
Effects of indoor and outdoor air pollutants on immune and nervous system. Susceptibility during critical windows and involvement of oxidative stress and inflammatory pathways are reported.

The contribution of this narrative review is to provide a comprehensive overview of the current knowledge about air pollution, with a particular focus on outdoor pollutants and PM, being the major public concern, and its impact on the immune and nervous system. Air pollution is a worldwide issue and its noxious effects on these two systems are not yet well characterized. We highlight the major findings to fill the existing lack of knowledge, mainly regarding the mechanism underlying the effects on the immune and, above all, nervous systems. Another contribution is to deepen the knowledge about the possible interaction between the immune and nervous system, proposing a general view and a link between the two systems, highlighted by the proposed mechanisms in Figure 1. To do this, we have taken into consideration literature reviews and original articles, using PubMed and Google Scholar, and we have selected key information about the effects on immune and nervous system, starting from epidemiological evidence, through in vivo and in vitro studies, to dissect the underneath mechanisms of action. Examples of keywords searched were “air pollution”, “particulate matter”, “ozone”, “diesel exhausted particles”, “nitrogen dioxide”, “carbon monoxide” combined with “immune system” and/or “central nervous system”. Our final aim is to have a general view of air pollution, to bring out the need to conduct further studies to better characterize this global problem.

## 2. Air Pollution and the Immune System

### 2.1. Lung and Skin: Route of Exposure

The intake and the uptake represent the two major processes by which a chemical can cross the body from outside. Intake is typically associated with inhalation, eating, or drinking [31], whereas uptake occurs by dermal contact, through skin absorption.

Air pollutants, such as SO_2_, NO_x_, CO, PM, and heavy metals, affect the respiratory system and a positive association between air pollution and lung cancer risk and mortality has been demonstrated in several epidemiological studies, particularly in individuals with existing lung disease [32,33]. An increase in alveolar protein concentration with an excess of ROS production has been associated with the exposure to UFPMs that can lead to elevated levels of oxidatively damaged DNA in culture cells, animals and humans [34].

PM is one of the most common air pollutants associated with an increased risk of exacerbation and respiratory symptoms [33]. In particular, PM_2.5_ and PM_0.1_, that are effectively respirable, have the potential to reach the alveoli [35,36,37]. Human exposure to PM may negatively influence lung activity. Studies conducted in various occupational workplaces suggested that PM could impact pulmonary function with sufficient evidence leading to assert that exposure to PM may cause lung cancer [23,38].

The second process by which a chemical can cross the human body is the uptake that involves skin absorption. Skin is a dynamic barrier of the human body able to maintain the internal homeostasis through neuropeptides, cytokines, hormones, and other effector molecules of the endocrine, immune, and nervous systems [39]. In industrialized countries, hypersensitivity reactions are, by far, the most frequently reported immune-derived effects of chemicals in humans [40]. Major air agents which affect the skin are solar ultraviolet radiation (UV), PAHs, volatile organic compounds, NO_2_, PM, cigarette smoke, O_3_, heavy metals, and As. Air contaminants can bind the stratum corneum, may permeate the epidermal barrier, and may enter the systemic circulation [41]. In their passage through the epidermis, they can be metabolized giving rise to reactive metabolites. The structural and functional integrity of the epidermis may be directly damaged or impaired through indirect phenomena, including inflammation [42]. A depletion of enzymes such as glutathione peroxidase and reductase, superoxide dismutase, and catalase, but also of non-enzymatic antioxidant complexes such as vitamin E, vitamin C, and glutathione, may contribute to modulate the exacerbation of oxidative stress reactions [43]. This persistent state of inflammation associated to air pollution has a fundamental role in the pathogenesis of both metabolic and immune-mediated diseases, causing primarily skin aging, allergic contact dermatitis, atopic dermatitis (AD), psoriasis, acne, and skin cancer [13]. Therefore, lung and skin are two of the mostly involved systems due to the primary route of exposure for air pollution.

### 2.2. Air Pollution Pathways in the Immune System

The increasing evidence that the immune system can be the target of many chemicals including environmental contaminants and drugs with potentially adverse effects on the host’s health has raised serious concerns within the public and regulatory agencies. Among air pollutants, a substantial amount of evidence has emerged from literature research regarding the toxic effects of PM and DEP. Bartra and colleagues [44] reviewed the overall effects of DEPs on the immune system: DEPs induced an enhancement of IgE-mediated air-allergen sensitization [45], Th2 cytokine response [46], increased the eosinophilic inflammation [47], and increased the production of IL-6 and IL-10 in patients with mild asthma [48]. It has also been demonstrated that DEPs induce a decrease in CD25 expression, IL-2, and IFN-γ in CD4+ and CD8+ T cells causing defective immune-surveillance, defective Th1 profile, and an abnormal persistence of activated T cells [49]. Furthermore, DEPs suppressed cell-mediated cytotoxicity and decreased the markers of cytotoxic natural killer cells [50]. This evidence strongly supports the hypothesis that DEPs exposure may increase the susceptibility to infections, having an immunosuppressive behavior. 

Consolidated data well demonstrated that DEPs mediate specific biological effects through the mitogen-activated protein (MAP) kinase cascade proteins activation (i.e., ERK, p38), and consequently with nuclear factors activation, such as NF-κB [51,52], Nrf2, and AP-1 [53,54,55]. Notably, comparable to other forms of PM, DEPs have been associated with oxidative stress, which can ultimately be related to genotoxic effects. In fact, several studies reported that exposure to DEPs is associated with increased levels of DNA oxidative damage in cultured cells detected as formamidopyrimidine DNA glycosylase (FGP)-sensitive sites by the comet assay [56]. 

A second outdoor air pollutant class is covered by PM. Oxidative stress-induced genotoxicity is thought to be an important link to carcinogenesis and has consistently been associated with PM exposure in experimental models [57,58]. PM rapidly increases ROS production, whereas the inflammation develops over time. This scenario has been demonstrated in vitro on a co-culture of human alveolar epithelial and macrophage cells: an increase in ROS production and pro-inflammatory markers (IL-1β, IL-6, IL-8 e TNF-α) following 24 h exposure to PM_2.5_ [59]. PM_2.5_ was also responsible for the induction of the p38 MAPK phosphorylation [59]. The involvement of MAPK and NF-κB pathways was also demonstrated by Al Hanai and colleagues [60]. PM_10_ was able to stimulate macrophage inflammatory pathways, inducing lung inflammation [61]. 

Another recent study focused on long-term air pollution exposure and DNA methylation showed a significant association of NO_2_ exposure with blood DNA methylation and lung function changes [62]. The authors reported a study conducted on volunteers exposed to NO_2_ and results showed a lung function decrease associated with DNA methylation. Interestingly, Clifford and colleagues [63] reported that exposure to DE may play a role in the development and progression of allergic disease, in particular allergic respiratory disease, suggesting that specific exposures can trigger the lung for changes in DNA methylation. 

As we previously described, skin is one of the first targets of air pollutants, due to their uptake. Exposure of the skin to air pollutants may lead to damage of proteins, lipids, and DNA, through the production of ROS and RNS. In the skin, air pollutants are able to cause a redox imbalance between oxidants and antioxidants through a direct damage and they lead to an increase in cutaneous levels of ROS that activate the release of pro-inflammatory factors and stimulate neutrophils activation and phagocytic cells, auto-feeding the pollutant response [41,64]. Furthermore, UVA could induce photo aging through the generation of ROS in keratinocytes and fibroblasts, which leads to the intracellular stimulation of kinases, transcription of nuclear transcription factors AP-1 and NF-κB [65]. UVA and UVB have been implicated in cutaneous immunosuppression and in photo-carcinogenesis after a mechanism of damage of structure and function of the cells due to a cascade of oxidative events [66,67]. 

It has been extensively reported that PM_2.5_ is able to penetrate the skin generating oxidative stress mainly through the conversion of the PAH, absorbed on the surface of PM, into quinones that explain a redox activity in the cells with the final production of ROS. It has been demonstrated their ability to decrease DNA methyltransferase and increase DNA demethylase, leading to skin senescence [68]. PM_10_, NO_2_, NO_x_ and CO have been associated with development and aggravation of AD in children and adolescents [69,70]. AD is a chronic relapsing inflammatory skin disease mostly occurring in early childhood and it is associated with other atopic disorders, such as allergic rhinitis and asthma [70]. The pathogenesis of AD involves both skin barrier defects and immunologic dysregulation. AD is still increasing in both developing and developed countries and indoor and outdoor air pollutants have been considered potential risk factors for the development or exacerbation of AD [42].

Another air pollutant able to affect the immune system is traditional cigarette smoke, a highly complex aerosol composed by thousands of chemical substances (e.g., ROS, CO, RNS, and electrophilic aldehydes) [71,72]. Traditional tobacco smoking causes premature aging [73] and an increased risk of psoriasis, acne, and skin squamous cell carcinoma [74]. It has been reported that exposure to traditional tobacco smoke, both active and passive, lead to the development of AD [75].

O_3_, a ubiquitous pollutant in urban environment, is able to damage the barrier of epidermis inducing the formation of peroxides, aldehydes and lipids ozonation [76]. In human keratinocytes O_3_ lead to an increase ROS formation and IL-8 gene expression [77]. From the reviewed papers emerged that the exposure to air pollutants is mainly associated with ROS production, inflammation, and oxidative damage to DNA, and in turn, these issues lead to adverse outcomes on the immune system and related organs. 

In sum, air pollutants play mainly a pro-inflammatory role [78] that could include direct oxidative effects of O_3_ or induction of ROS. Some variability of these effects was shown among the different exhausts and particles investigated, suggesting that distinct physiochemical properties could arise from different materials generated and collected in specific conditions. Between the epigenetic mechanisms that may contribute to inflammation reaction and lung injury, such as asthma, DNA methylation is one of the most studied [79], but still requires more attention and investigations.

### 2.3. Air Pollution in Immune System Critical Windows: Pregnancy, Infancy and Childhood 

Air pollution each year causes approximately one third of premature deaths [80]. Exposure to air pollutants during prenatal period can affect the growth during infancy and childhood [81] acting mainly through the impairment of organogenesis. Regarding the immune system development, the period from in utero to the first years of life is a critical time. The maturation of the immune system can be influenced if exposed to environmental pollution in early life [82]. For this reason, the effects of the exposure to toxic pollutants during this period could arise in an increased risk of allergies later in life [83]. In particular, the most sensitive time windows are pregnancy, infancy, and childhood.

PM exposure during pregnancy can lead to a delayed maturation of the immune system of the fetuses and this can give them a higher vulnerability to allergies development [84]. PM effects on the immune system could be mediated by toll-like receptors (TLRs) [85]. The study of Becker et al. [86] revealed higher levels of TLR2 and TLR4 upon exposure to PM_2.5–10_. TLRs are essential for the immune response of newborns and, for this reason, a dysregulation of them could have an impact on the immune system [87]. A study performed in the Czech Republic highlighted the effect of air pollutants exposure during gestation on the fetal immune system, indicating that, above all, PAHs and PM_2.5_ can act as immunomodulators impacting on infant and children health [88]. There are several studies that have investigated the effects of air pollution during pregnancy, and some of them have reported associations with allergies and lower respiratory tract infections early in life. In a study conducted in a cohort of 5733 children in Wuhan, China, prenatal and early-life exposure to PM less than 1 μm in diameter, PM_2.5_, and PM_10_ were associated with increased risk of development of asthma by the age of 3 years [89]. Another prospective cohort study revealed that parental exposure to NO_2_ had significative associations with allergic diseases in childhood only in sensitive windows; for example, correlations between asthma and the second trimester and between allergic rhinitis and the third trimester were assessed [90]. A further study conducted in Canada reported an association between NO_2_ and PM_2.5_ exposure in the second trimester with an increased risk of asthma development in children [91]. A study conducted on mice also revealed that O_3_ exposure during the gestation period could lead to airway inflammation and, particularly, it has been shown to act through the imbalance of the Th1/Th2 differentiation in the offspring, increasing the severity of asthma [92]. Kannan and colleagues [93] demonstrated that inhaled pollutants by pregnant mothers can decrease the nutrient/oxygen supply, through inflammatory and immune reactions of the mother, or can act directly on the fetus, crossing the placenta. Three critical periods of immune system development during the gestation period were identified: the first phase is the initiation of hematopoiesis (week 8–10), the second phase is the migration of hematopoietic cells and the expansion of progenitor cells (weeks 10–16) and the last period is the colonization of thymus and bone marrow (weeks 16-birth). The immune system is not fully mature even after birth; the maturation to the immunocompetence lasts until the year of age and the establishment of the immune memory is not completed until the 18 years [94]. For these reasons, air pollution exposure during all these phases could result in immune system alterations.

Regarding infancy, it is a high-risk period for allergic sensitization because the immune defenses are under development. Allergy prevalence has increased over the years, and above all in children. One reason of this enhancement could be the exposure to air pollutants [95]. The environmental exposure during early post-natal life could determine the development of allergies during the entire lifespan and above all during childhood [96]. Fetuses and infants have immature immune and respiratory systems and for this reason they are more susceptible to air pollution effects. In their systematic review on the association between traffic-related air pollution (mainly PM_10_, PM_2.5_, NO_x_) and their effects on children, authors highlighted a connection between NO_2_ and PM_2.5_ exposure during early childhood and increased asthma and food sensitization incidences [94]. Furthermore, there is some evidence of association also between traffic-related air pollution and eczema [97]. Other associations, highlighted in the literature, connect NO_2_ exposure during early life and both asthma and AD prevalence during childhood [98,99]. 

As infants, children are also vulnerable to air pollution, since they are characterized by a higher ventilation rate and narrower airways than adults, they spend more time outside and their immune system is not fully mature [100]. A study conducted in California on 188 children (median age: 14.7 years), of which 35.6% were asthmatics, investigated the correlation between exposure to high levels of CO, NO_2_, and PM_2.5_ and the alterations in differentially methylated regions of genes involved in the immune system and asthma. It has been revealed that air pollutants exposure was associated to an altered profile of methylation of FoxP3 and IL-10 [101]. Other evidence comes from studies based on the traffic-related air pollutants, which were associated with atopic diseases, allergies, and asthma in schoolchildren [102,103]. Regarding the mechanisms, lung diseases due to air pollution can be mediated by the induction of an inflammatory state [26]. The short-term increase in the environment of black carbon, DEPs, and NO_2_ induced increased airways inflammation and oxidative stress in a pediatric population [104]. In fact, pollutants can lead to ROS production, and are able to damage cells and react with DNA and lipids [105]. Therefore, the immune system is widely affected by air pollutants, as reported in Table 2, and above all, it results in being more susceptible to air pollution and PM during the above-mentioned critical windows, leading to a higher vulnerability to infections and allergies.

## 3. Air Pollution and the Nervous System

### 3.1. Air Pollution Way to the Brain

The fine and ultrafine fractions of PM (PM_0.1_ and PM_2.5_) are predominantly implicated in CNS effects, given their capability to reach the brain. PM is inhaled on a regular basis due to air pollution, crosses the alveolar-capillary barrier in the lungs, thus gaining access to peripheral circulation and distributing to various organs, including the brain [106]. Active transport, a leaky blood–brain barrier (BBB), and translocation through the nasal olfactory pathway have been proposed as mechanisms by which the PM enters the brain. Air pollutants (e.g., Al nanoparticles-NPs- of 8–12 nm diameter size) could damage the endothelial cells of the BBB and alter its protective functions [107], distributing the systemically delivered PM_0.1_ to the brain parenchyma. Differently, nasally inhaled pollutants deposited on the olfactory mucosa, can be subsequently translocated via the olfactory nerve to the olfactory bulb [108], which is an accumulation site of NPs. In addition, CNS distribution could occur in the piriform cortex, olfactory tubercule, amygdala and, through anterograde pathways, could reach the cortex, thalamus, hypothalamus, hippocampus, striatum, and cerebellum [109,110,111], as demonstrated by exposing rats via whole-body exposure to Mn oxide NPs, a metal associated to industrial-derived air pollution. Air pollutants do not need to physically reach the brain to provoke an adverse effect: they could induce oxidative stress and trigger the release of soluble inflammatory mediators from primary entry organs or secondary targets [21]. Those mechanisms were suggested for O_3_, which is rapidly consumed in the lungs because of its reactivity, and mediate extrapulmonary toxicity through secondary products released in the blood [112]. Finally, air pollution exposure induces the activation of hypothalamic-pituitary-adrenal axis with a consequent stress response, which is a feature of many disease processes [113]. Therefore, there are different ways through which air pollutants could reach the CNS, with the possible consequent associated adverse outcomes.

### 3.2. Air Pollution in CNS Critical Windows: Neurodevelopment and Neurodegeneration

Epidemiological studies have pointed out the connection between air pollution and brain, because an increasing number of papers affirm that air pollution exposure may be associated with a brain deficit concerning neurodevelopment, such as behavioral and cognitive deficits, psychiatric disorders, depression, and autism [114,115,116,117,118] and with an increased incidence of childhood CNS tumors [119]. 

Air pollution influences CNS development, and young individuals seem to be particularly susceptible to air pollution-induced neuronal damage starting from the prenatal period, when air pollution exposure might cause, or contribute to the development of disabilities and behavioral deficits [84] by disturbing postnatal brain growth and by interfering with its maturation, altering neurogenic/gliogenic events, myelination, and synaptogenesis [120]. 

An Italian study in the Milan area showed that PM_10_ exposure, during the first trimester of pregnancy, is negatively associated with weight at birth [121]. Clinical studies performed in children living in Mexico City and exposed to high levels of air pollution (O_3_ and PM) report structural damage at the prefrontal cortex, as well as elevated neuroinflammatory markers that have been, potentially, associated with cognitive deficits [122,123,124]. Furthermore, different studies have revealed an association between air pollutants, related to elemental carbon attributed to traffic, and the occurrence of hyperactivity [125]. Moreover, children prenatally exposed to high levels of PAH showed anxious/depressive symptoms at 6–7 years of age [126] thus supporting the idea that air pollution exposure in early life could trigger diseases later in life, after a time delay, possibly due to fetal re-programming at the molecular level of biological features. 

Such epidemiological evidence is supported by in vivo studies on rodents. In utero exposure to DE alters locomotor activity, motor coordination, and causes impulsive behavior in male mice, with concomitant alterations in dopamine turnover and monoamine metabolism in a variety of brain regions [127,128]. An additional in vivo study showed that prenatal NPs-rich DE exposure to female mice caused an altered spatial learning and memory [129]. Furthermore, the exposure to nanosized PM to adult female mice induced an impaired differentiation of cerebral cortex neuron of the offspring [130]. From a behavioral point of view, prenatal and early life exposure of mice to DEP is associated with reduced social interaction, increased repetitive behaviors, and reduced or altered communications [131]. 

Together with children, the elderly mostly seem to be susceptible to air pollution. Such exposure, during aging, may contribute to the onset of neurodegenerative disorders, enhancing the progression of neurodegenerative processes [132]. Analyses of post-mortem tissues from humans living in highly polluted areas have shown an increase in beta-amyloid 42, hyper-phosphorylated tau and alpha synuclein, which are potential markers of pre-clinical stages of Alzheimer’s and Parkinson’s diseases. Moreover, a dose–response relationship between air pollution exposure and test performances was found in individuals with increased incidence of mild cognitive impairment [133,134].

An interesting study conducted in the Italian area of Val Camonica showed an increased prevalence of deficits in coordination and cognitive abilities, related to the high Mn levels in PM present in the deposited dust from ferroalloy airborne emissions [135]. These results match with the in vivo data on rat brain showing an increase in pro-inflammatory mediators, markers of oxidative stress, and immune cells activation after Mn oxide NPs exposure, indicating that the olfactory neuronal is involved in the translocation of this NP to the CNS [110]. Together, these findings suggest that air pollution may potentially impact neurodegenerative pathways (for an extensive review about air pollution and dementia see [136]) and that the elderly are more vulnerable to air pollution because the aged BBB becomes more penetrable or because the damage accumulation due to a chronic exposure throughout the entire life [137]. 

Thus, neurodevelopment and neurodegeneration are two critical time windows in the CNS, during which the brain has an increased susceptibility to air pollution exposure-related outcomes.

### 3.3. Air Pollution Pathways in the CNS

Although the exact mechanisms driving brain pathology induced by air pollution are not completely understood, oxidative stress and inflammation are pointed out as common features triggering air pollution neurotoxicity [138]. Evidence of the involvement of these pathways comes from human studies. Brain tissues from individuals residing in highly polluted areas showed an increase in infiltrating monocytes or resident microglia [139]. Increase in pro-inflammatory mediators such as IL-1β, COX-2, and CD14 marker for innate immune cells were observed in frontal cortex, substantia nigra, and vagus nerve [139]. 

Air pollutants also induce a neuroinflammatory response in vivo. Mice exposed to DE exhibited microglial activation and, consequently, a sex dimorphic impairment of adult neurogenesis with males showing fewer neurons in the olfactory bulb, hippocampal sub-granular zone, and sub-ventricular zone, while females only showed fewer neurons in the olfactory bulb [140]. Further, 6 h/day and 5 days/week exposure of mice (from embryonic day 0 to postnatal day 21) to DE at the level of 250–300 µg/m^3^ induces an increase in IL-6 in placenta and in neonatal brain [141]. Both prolonged and short-term exposure of rodents to DE enhanced the production of several pro-inflammatory and oxidative stress markers in different regions of the brain [142,143,144,145].

These findings rest on previous evidence obtained by in vitro studies, which demonstrated that microglial cells are activated by DE, resulting in morphological changes, increased superoxide, and ROS production. In neuron–glia cultures, DEP selectively damaged dopaminergic neurons that only occurred in the presence of microglia, indicating that microglia mediate the DEP neurotoxicity [146]. Neuron–glia cultures derived from mice lacking nicotinamide adenine dinucleotide phosphate oxidase, the enzyme responsible for microglial extracellular superoxide production, were insensitive to DEP-induced neuronal damage, indicating that microglia-derived ROS are crucial for DEP-induced dopaminergic neurotoxicity [146]. Moreover, microglia exposed to concentrated ambient air particles increases the expression of pro-inflammatory mediators, such as IL-6 and TNF-α [147], suggesting that some forms of PM induce cytokine production and modulation of inflammatory pathways. In astrocytes and microglia cultured together and exposed to PM (diameter <0.2 µM) a strong activation of TLR4 and NF-κB has been observed, suggesting that TLR4 mediated inflammatory pathway is central to the brain response to air pollution [148]. 

Other cells of the CNS are also a target of detrimental effects induced by air pollution exposure. It was first reported that astrocytes are activated in humans chronically exposed to high levels of air pollution [149]. More recently, the effects of air pollutants on astrocyte–microglia crosstalk were described, with a particular focus on pro-inflammatory activation and oxidative stress [150]. However, it is not clear if the activation is due to the components of air pollution, or to the inflammation and oxidative stress produced from other cell types, or to cellular damage. 

Moreover, oligodendrocytes, which in the past years have been largely overlooked in air pollution research, are altered after air pollutant exposure. The few papers available demonstrated the presence of apoptotic glial white matter cells in dogs in Southwest Metropolitan Mexico City, a highly polluted urban region [151], and an alteration of white matter integrity in children resident in Mexico City and, thus, chronically exposed to significant concentration of O_3_, PM and PM-associated lipopolysaccharide [133]. Recent in vivo findings provide the first experimental evidence of the reduction in myelin basic protein after air pollution exposure [148]. This evidence in mice is consistent with the study of Chen and colleagues [152] which showed, in old women with greater exposure to PM_2.5_, a significant reduction in white matter, but not gray matter volumes and with previous research by Peterson and collaborators [153] which found that higher prenatal PAH exposure corresponded with reductions in white surface matter of children brains. 

In addition, air pollution could also directly affect neuronal cells: in primary hippocampal neurons, PM_2.5_ elevated COX-2 expression, through NF-κB modulation, and in hippocampal brain slices, increased the amplitude of field excitatory postsynaptic potentials, intracellular ROS generation, glutathione depletion, and loss of mitochondrial membrane potential [154]. In conclusion, several nervous system components can be affected by different air pollutants, as reported in Table 3.

**Figure 2:**
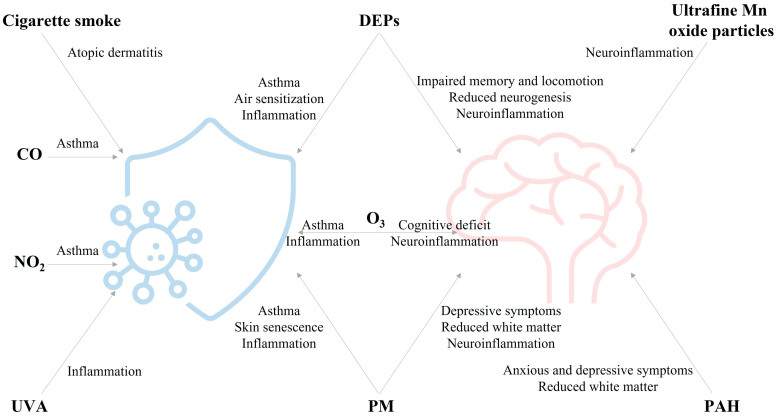
Main effects of air pollutants on immune and nervous system. Summary of the results reported in Table 2 and Table 3.

A summary of the main adverse effects posed by air pollutants on the immune and nervous system is provided in Figure 2.

## 4. Possible Interaction between the Immune and Nervous System?

### 4.1. Neuro–Immune–Cutaneous–Endocrine (NICE) Network

As mentioned before, large epidemiological and observational studies identified that both long- and short-term exposure to airborne pollution increase adverse health outcomes, such as hospitalization, morbidity, and mortality. It is well known that exposure to air pollutants has been associated with marked increases in cardiovascular disease, such as myocardial ischemia, arrhythmia, heart failure, and respiratory disease, such as lung cancer and asthma. Recently, a link between particle pollution and the occurrence of neurological disease has also been indicated, such as in cognitive dysfunctions, and Alzheimer’s and Parkinson’s diseases [155]. In addition, there is increasing evidence that environmental pollution may exert negative effects on human skin that acts like an interface between the body and the surrounding atmosphere and is therefore the primary contact for environmental pollutants [156].

A number of mechanisms have been proposed to explain the adverse health impact of air pollutants, and in particular PM, such as inflammation [157], production of white blood cells [158], ROS and RNS production in the lungs [159], endotoxin-mediated cellular and tissue responses, stimulation of irritant receptors, and covalent modification of key cellular enzymes [160]. ROS can damage cellular proteins, lipids, membranes, and DNA, and lead to an increase in the production of antioxidant enzymes through activation of the transcription factor Nrf2 [161]. Failure to overcome oxidative stress leads to the activation of additional intracellular signaling cascades that regulate the expression of cytokine and chemokine genes that imply pro-inflammatory effects, locally but also systemically.

The pathobiology likely begins principally in the lungs and in the skin, but how does a pulmonary/skin event translate to a systemic and/or central event? Skin should be considered a sensory organ, due to the cutaneous innervation as part of the peripheral nervous system, and also is an important part of CNS, as active interface and first connection of the body to the external body insults [162]. Connection between nerve fibers, immune, endocrine, and cutaneous cells have been demonstrated in skin and it has been identified with the acronym NICE, the “neuro–immune–cutaneous–endocrine” network [163,164]. NICE system shares a language of neuropeptides, cytokines, glucocorticoids, potential growth factors, hormones, and other effector molecules in a bi-directional way, from the brain to the body and vice versa [165]. In particular, Langerhans cells (LCs), acting as antigen presenting cells, express some neuronal markers and neuropeptide receptors, and are closely connected to the nerve fibers in the epidermis. Thus, LCs and nervous system are anatomically and functionally connected, and Misery and colleagues [166] suggested that they belong to a NICE system.

What clearly emerged from the literature research about mechanisms mostly involved in the health effects of air pollution is the induction of an oxidative stress status due to a redox imbalance arising from either the overproduction of oxidants or an impaired antioxidant defense system. It is postulated that a significant PM-induced pulmonary inflammatory response releases pro-inflammatory cytokines into the circulation, which are enough to produce a systemic response [59,167]. Ambient particle pollution might not only affect healthy skin with a statistically significant increase in urticaria, eczema, contact dermatitis, rush but additionally can exert detrimental effects in diseased skin, such as in the case of AD [167]. Current research suggests that air pollutants have two different toxic modes of action: (i) the outside–inside or (ii) the inside-outside mechanisms. The first one is based on the penetration of the pollutants into the skin with the consequent exacerbation of the toxic activity locally and systemically. Regarding the second mechanism of action, after the lung penetration, particles could generate inflammatory reaction locally, but can also cause systemic inflammatory reactions, passing through the circulation. The systemic inflammation induced by air pollutants could affect the skin and other districts, including the CNS [168,169]. Circulating inflammatory mediators, derived from pollutant inflamed organs, may activate peripheral neuronal afferents and gain access to the brain by passive diffusion at the circumventricular organs, which are brain areas lacking the BBB, or active transport across the BBB to impact on the CNS [170,171]. Engagement of these immune-to-brain communication pathways ultimately leads to the subsequent activation of the brain’s immune surveillance cells (e.g., microglia) giving rise to a local inflammatory response and enhancing the production of pro-inflammatory cytokines [172]. These events could alter the CNS immune response, neuronal functions, and/or behavior. Besides cellular damage and induction of inflammatory mediators in the brain, systemic inflammation alters the cellular make-up of innate immune cells in the brain. In particular, studies in animal models have highlighted a role for TNF-α, IL-1β, and IL-6 in mediating the communication between the periphery and the brain during systemic inflammation through the recruitment of a large number of circulating monocytes [173]. Furthermore, cytokines could cause injury to cerebral vascular endothelial cells, compromising the integrity and the protective role of the BBB, or triggering signaling cascades that lead to the activation of the MAP kinase and NF-κB-mediated pathways. Disruption of BBB integrity could be followed by the trafficking of mast cells and inflammatory cells to the damage sites [133]. Thus, systemic inflammation caused by air pollution could give rise to both neuroinflammation and neuropathology, where neurotoxic effects may be cumulative. 

### 4.2. Role of Microbiota: The Hygiene Hypothesis

A link between air pollution and human microbiota could be expected. The higher incidence, mainly in industrialized countries, of several diseases, namely allergies, autoimmune disorders, CNS diseases, and cancer, could be linked to the hygiene hypothesis, which proposes that certain microorganisms can protect the host against inflammatory diseases and that their alteration can be the cause or, better, an exacerbating factor for the development of various health problems [174,175].

Human microbiota colonizes the human body, and mainly resides in gut, skin, lung, and vagina. In certain disorders where environmental factors, such as air pollution, are implicated, an imbalance between commensal bacteria could have an important role in pathogenesis [176]. The hygiene hypothesis suggests that decreased airway microbiota diversity is linked to an increased asthma prevalence [177]. Indeed, it is known that lung microbiota plays a pivotal role in health and disease [178]. Not only is the lung microbiota involved, it has been demonstrated that airborne PM exposure alters the gut microbiota too and induces acute and chronic inflammatory responses in the intestine [179,180]. A study on mice revealed that inhalation exposure to PM_2.5_ induced changes in the bacterial composition throughout the gastrointestinal tract, parallelly with an increased inflammatory response [181]. Recently, UFPMs also revealed the ability to change gut microbiota composition in a mouse model [182]. 

Regarding the connection between air pollution, microbiota, and CNS, an important player is the gut–brain axis. The gut microbiota is also called the “second brain” [183]; to date, it is known that the microbiota influences neuronal activity as demonstrated by single-nucleus RNA sequencing experiments which revealed significant alterations in gene expression in excitatory neurons, glia, and other brain cell types in antibiotic-treated or germ-free adult mice [184]. The above-mentioned effects of air pollution on gut microbes reflect on CNS, mainly through the involvement of the immune system [185]. Microglia, for example, play a pivotal role in the microbiota gut–brain axis, with several implications for neurodegenerative disorders [186]. Moreover, a combination of probiotic bacteria was demonstrated to improve emotional behaviors in animals and psychological outcomes in rodents and humans [187,188], thus microbiota alterations due to air pollution exposure could contribute to mental illness.

Due to the novelty of this field of research, it is mandatory to better investigate the role of microbiota in air pollution-related CNS and immune effects.

## 5. Future Directions and Other Considerations

Given the high number of deaths per year ascribed to outdoor air pollution (e.g., 4.2 million), it is important to deepen the actual knowledge regarding its effects, and it is mandatory to highlight that 90% of people live in places with an air quality that does not meet WHO guideline limits [6]. Public policy has a central role in lowering air pollution mainly through the reduction of emissions and setting national standards that meet WHO air quality guidelines. Invest in research and education focused on unveiling the mechanistic data underneath air pollution systemic effects, represent an essential tool. Some examples of interventions to reduce air pollution include developing sustainable transport in cities; implementing solid waste management; providing access to clean household fuels and cook stoves; developing market for renewables energies, and energy efficiency and implementing industrial emissions reductions [189]. Furthermore, it is important to mention the role played by WHO, for example, in the development of tools such as AirQ+, a software able to perform calculations that allow quantification of the health effects of exposure to air pollution; the Health Economic Assessment Tool (HEAT) to assess walking and cycling interventions; the Green+ tool to raise importance of green space and health; the Sustainable Transport Health Assessment Tool (STHAT) and the Integrated Transport and Health Impact Modelling Tool (ITHIM); and the Clean Household Energy Solutions Toolkit (CHEST) to provide countries and programs with the tools needed to create or evaluate policies that expand clean household energy access and use [11]. Moreover, in Europe the global fight against air pollution is today stronger than ever and in 2021, the European Commission adopted the EU Action Plan: “Towards a Zero Pollution for Air, Water and Soil”—a key deliverable of the European Green Deal. The “zero pollution” vision for 2050, divided in several steps, actions, and targets, is to create a toxic-free environment, achieving climate neutrality [190]. To mitigate the air pollution problem, many efforts must be taken not only by national governments but exposure can also be reduced by personal choices. In fact, a lifestyle change adopted by each citizen, may contribute to the mitigation of air pollution [191]. Some practical examples of these behavioral changes that people should easily apply are to use of the public and the active transport instead of private vehicles [192,193]; use of facemasks under appropriate circumstances [194]; optimize driving style and vehicle settings [195]; and moderate outdoor physical activity when and where air pollution levels are high [196]. In addition, the balance of personal risks and benefits due to the exposure to air pollutants could be optimize considering the individual sensitivity [194] considering that fetuses, children, the elderly, and individuals affected by advanced or chronic diseases represent the most susceptible categories to clinical health problems related to air pollution [197,198,199,200]. The main goal of all these guidelines, recommendations, and programs is to provide guidance able to reduce air pollution levels and subsequently to decrease the huge health burden resulting from exposure to air pollution worldwide.

## 6. Conclusions

As defined by the WHO, air pollution is a contamination of the indoor or outdoor environment by any chemical, physical, or biological agent that modifies the natural characteristics of the atmosphere. PM, CO, O_3_, NO_2,_ and SO_2_ are the major components of outdoor air pollution and are responsible for a large proportion of health-related pathologies. In this review, we highlighted the major findings regarding the effects of air pollutants on the immune and nervous systems, also investigating their mode of action.

In this context, it is of note that human beings are exposed simultaneously to mixtures of air pollutants and not to single entities. Therefore, the human adverse health outcome should be investigated, taking into account multiple substances. In the literature, only a few recent studies investigated the effects of air pollutant mixtures and health effects [201,202,203,204,205]. Recently developed methodologies able to evaluate the presence of multiple chemicals can also be applied in the air pollution field [206] and statistical approaches to estimate multi-pollutant mixtures or multiple correlated exposure effects on human health are an important area of ongoing research, also for regulatory purposes (reviewed in [207]).

Due to the complexity of air pollution composition, the mechanisms involved in organ dysfunctions and pathologies are not completely clear. Nevertheless, oxidative stress and ROS production play a key role in the induction of primary inflammation. Through the activation of downstream inflammatory pathways, that finally led to an increase in cytokines expression and to immune cells activation, inflammatory status results are self-maintained. An integrated view of the brain and skin relationship is leading to a postulation of the NICE network that could be conceived, such as a bridge between the brain and the skin composed by cutaneous cells (melanocytes, keratinocytes, fibroblasts, etc.), cutaneous immune cells (LCs, lymphocytes, and macrophages) and cutaneous nerve fibers. A crosstalk between all these cells occurs through the release of neuropeptides, cytokines, hormones, and other effector molecules.

In this context, our review aimed to provide a general view of the interconnections among air pollution, CNS, and immune system shedding light on crossing aspects between the two systems, which are usually overlooked. In the literature, we found extensive data regarding outdoor air pollutants, namely DEP and PM; conversely, little information regarding other pollutants and in general indoor pollution is present. Therefore, there is an urgent need of research on this area. Differently from what exists in the literature, we highlighted the common mechanisms between the immune and nervous systems and the link between them. Hence, with this review, we have summed up the effects of air pollution and the possible mechanisms, but the action linking to the pathological outcome remains unclear, thus deeper investigations are required.

## Figures and Tables

**Table 1:** Summary of recommended Air quality guideline (AQG) levels. Adapted from WHO Global air quality guidelines—2021 [19].

Pollutant	Averaging Time	AQG Level
PM_2.5_ μg/m^3^	Annual	5
	24 h ^a^	15
PM_10_ μg/m^3^	Annual	15
	24 h ^a^	45
O_3_ μg/m^3^	Peak season	60
	8 h ^b^	100
NO_2_ μg/m^3^	Annual	10
	24 h ^a^	25
SO_2_ μg/m^3^	24 h ^a^	40
CO mg/m^3^	24 h ^a^	4

^a^ 99th percentile (e.g., 3–4 exceedance days per year); ^b^ Average of daily maximum 8-h mean O_3_ concentration in the six consecutive months with the highest six-month running average O_3_ concentration.

**Table 2:** Summary of air pollutant effects on the immune system.

Air Pollutant	Effect	Type of Study	References
Active/passive cigarette smoke	Increased risk of AD.	Systematic review and meta-analysis	[75]
CO	Modified methylation of FoxP3 promoter and IL-10 leading to asthma.	In vivo	[101]
DEPs	Enhanced IgE mediated air-allergen sensitization.	Murine model	[45]
	Enhanced Th2 cytokines response.	Asthma murine model	[46]
	Eosinophilic inflammation.	Guinea pigs sensitized to pollen	[47]
	Increased production of Il-6 and IL-10 in patients with mild asthma.	Asthmatic patients	[48]
	Decreased CD25 expression, IL-2 and IFN-γ in CD4^+^ and CD8^+^ T cells.	In vitro	[49]
	Decreased release of IL-1β, IL-2, IL-4, IL-12p70, IFN-γ, and TNF-α by NK cells.	In vitro	[50]
	Increase in ERK, p38, and NF-κB.	In vitro	[51]
	Increase in MMP-1 and ERK1–2 phosphorylation.	In vitro	[52]
	Activation of Nrf2.	In vitro	[53]
	Increase in nuclear translocation NF-κB, AP-1, phosphorylated Jun kinase, and phosphorylated p38.	Nonatopic non-smokers (in vivo)	[54]
	MAP kinase-mediated activation of NF-κB and AP-1.	In vitro	[55]
	Increased oxidative damage of DNA.	In vitro	[56]
	Changes in DNA methylation that increase the development and progression of allergic respiratory disease.	Randomized crossover-controlled exposure study	[63]
NO_2_	Induction of blood DNA methylation and lung function changes.	Cohort study	[62]
	Modified methylation of FoxP3 promoter and IL-10 leading to asthma.	In vivo	[101]
O_3_	Increased ROS formation and IL-8 gene expression in keratinocytes.	In vitro	[77]
	Imbalance of the Th1/Th2 differentiation in the offspring, increasing the severity of asthma.	In vivo	[92]
PM	Increase in ROS production, pro-inflammatory markers (IL-1β, IL-6, IL-8 e TNF-α) and phosphorylation of p38 MAPK.	In vitro	[59]
	Increase in ROS production, TNF-α and involvement of MAPK and NF-κB pathways.	In vitro	[60]
	Increased GM-CSF levels, MIP-1β, MCP-1, IL-6, and ICAM-1.	In vitro	[61]
	Decrease in DNA methyltransferase and increase in DNA demethylase leading to skin senescence.	In vitro	[68]
	Increased levels of TLR2 and TLR4.	In vivo	[86]
	Modified methylation of FoxP3 promoter and IL-10 leading to asthma.	In vivo	[101]
UVA	Induced ROS production in keratinocytes and fibroblasts, leading to the transcription of nuclear transcription factors AP-1 and NF-κB.	In vitro	[65]

**Table 3:** Summary of air pollutant effects on the nervous system.

Air Pollutant	Effect	Type of Study	References
Air pollution	Up-regulation of COX-2, IL-1β, CD14 in olfactory bulb, frontal cortex, *substantia nigra* and vagus nerve, and increase in infiltrating monocytes or resident microglia. Disruption of BBB, higher oxidative stress, and inflammatory mediators.	In vivo, children and young adults	[133,139]
DEPs	Alteration in dopamine turnover and monoamine metabolisms, leading to decrease in spontaneous locomotor activity.	In vivo, mice (in utero)	[127,128]
	Higher expression of NMDA receptor subunit GluN2A, CCL3, and BDNF indicating an impaired special learning and memory function.	In vivo, mice	[129]
	Increase locomotor activity and repetitive behaviors in offspring, referable to autism.	In vivo, mice (during pregnancy and nursing)	[131]
	Impaired cell proliferation only in males, reduction in adult neurogenesis, microglial activation, neuroinflammation and oxidative stress, indication of a sex-dependent impairment.	In vivo, mice	[140]
	Increased IL-6 release in the placenta and neonatal brain with the consequent activation of the JAK2/STAT3 pathway in neonatal brain.	In vivo, mice	[141]
	Increase in TNF-α and IL-1α in the striatum region.	In vivo, rats	[142]
	Increased CYP1A1, iNOS, and oxygenase-1 and COX-2 in different brain regions.	In vivo, rats	[143]
	Increase in IL-1α, IL-1β, TNF-α, IL-6 with some gender difference in olfactory bulb and hippocampus. Increase in oxygenase-1, nNOS and NMDA subunit GluN2A in male hippocampus.	In vivo, mice	[145]
	Dose-dependent decrease in dopaminergic neurons. Increase superoxide and ROS production.	In vitro	[146]
Elemental carbon attributed to traffic	Association with hyperactivity *T-*score.	In vivo, children	[125]
O_3_	Structural damage at prefrontal cortex, elevated neuroinflammatory markers associated with cognitive deficits.	In vivo, children	[122,123,124]
PAH	Increase in anxious/depressive symptoms.	In vivo, children	[125]
	Dose–response reduction in white matter.	Cross-sectional imaging study in school-age children	[153]
PM	Reduce weight at birth.	Prospective study in women during the first trimester of pregnancy	[121]
	Structural damage at prefrontal cortex, increase neuroinflammatory markers associated with cognitive deficits.	In vivo, children	[122,123,124]
	Altered neuronal differentiation in the offspring and depression-like symptoms in adult males.	In vivo, mice	[130]
	PM reached in Mn is associated with motor coordination and cognitive abilities deficits and increased levels of prolactin in serum.	In vivo, elderly human	[135]
	Reduces intracellular levels of ATP and increase in TNF-α and IL-6.	In vitro	[147]
	Activation of TLR.4 and NF-κB (in vitro). Increase in TLR.4, MyD88, TNF-α and TNFR2, and decrease in NF-κB in the hippocampus (in vivo).	In vitro/in vivo	[148]
	Decrease in white matter.	Prospective study in women	[149]
	Increase in COX-2 expression, ROS production and NF-κB phosphorylation.	In vitro	[154]
Ultrafine Mn oxide particles	Increased TNF-α, macrophage inflammatory protein-2, and neuronal cell adhesion molecules in olfactory bulb.	In vivo, rats	[110]
